# Somatic Embryogenesis of Immature *Cunninghamia lanceolata* (Lamb.) Hook Zygotic Embryos

**DOI:** 10.1038/s41598-017-00156-1

**Published:** 2017-03-03

**Authors:** Ruiyang Hu, Yuhan Sun, Bo Wu, Hongjing Duan, Huiquan Zheng, Dehuo Hu, Huazhong Lin, Zaikang Tong, Jinliang Xu, Yun Li

**Affiliations:** 1 National Engineering Laboratory for Tree Breeding, College of Biological Sciences and Technology, Beijing Forestry University, Beijing, 100083 China; 2 Guangdong Academy of Forestry, Guangzhou, 510520 China; 3 Fujian Jiangle State-owned Forestry Farm, Fujian, 353300 China; 4 The Nurturing Station for the State Key Laboratory of Subtropical Silviculture, Zhejiang A & F University, Zhejiang, 311300 China; 5 Kaihua Forestry Farm, Zhejiang, 324300 China

## Abstract

Two efficient somatic embryogenesis systems were developed in Chinese fir, the most important conifer for industrial wood production in China. Three development stages (cleavage polyembryony, dominant embryo, and precotyledon) of immature embryos derived from 25 genotypes of open-pollinated mother trees were used as initial explants. Cleavage polyembryony-stage embryos with a 12.44% induction rate was the most embryogenic response stage. The highest frequency of embryogenic callus (13.86%) induction was obtained from DCR medium supplemented with 1.5 mg L^−1^ 2,4-dichlorophenoxyacetic acid (2,4-D) and 0.3 mg L^−1^ kinetin (KN). An average of 53.33 early somatic embryos were produced from approximately 0.2 g (fresh weight) embryogenic callus after 2 weeks of incubation on medium supplemented with 50 μmol L^−1^ abscisic acid (ABA) and 100 g L^−1^ polyethylene glycol (PEG) 6000. About 53% dominant embryos have an embryogenic response after a 6-week cultivation on medium supplemented with 1.0–2.0 mg L^−1^ benzyladenine (BA), 0.2 mg L^−1^ naphthylacetic acid (NAA) or 2,4-D, and 0.004 mg L^−1^ thidiazuron (TDZ). After three successive transfer cultures on medium supplemented with 1.5 mg L^−1^ BA, 0.2 mg L^−1^ NAA, and 0.004 mg L^−1^ TDZ, 4.49–16.51% of the embryos developed into somatic embryos.

## Introduction

Somatic embryogenesis (SE) is defined as a process of differentiation of cells with a bipolar structure resembling a zygotic embryo into a plant. Somatic embryos that develop from a non-zygotic cell have no vascular connection with the original tissue^1^. More than 50 years ago, the initial somatic embryos were observed to develop from carrot cell suspensions^2^. Since then, the potential of SE has been shown in a wide range of plant species, including herbs^3^, liane^4^, shrubs^5^, and trees^6^. The unique developmental potential of SE represents a very powerful biotechnological tool for regenerating plants from cell culture systems, and it has also been recognized as a potential model for investigating the structural, physiological, and molecular events occurring during plant embryogenesis^7^. For conifers, since the first report of SE in Norway spruce in 1985^8^, this *in vitro* process has been initiated for a number of conifer species. SE is a multi-step regeneration process involving initiation, proliferation, maturation and plantlets regeneration. According to the occurrence mode, there are two pathways direct and indirect SE, and the main difference between them is with or without an intervening callus phase from induction to maturation when producing somatic embryos^1^. Somatic embryos were induced directly from an explant, or indirectly via an intervening callus phase, by manipulating the exogenous plant growth regulators in *Rosa hybrida* ^5^. In conifer SE, numerous studies have also successfully reported regeneration of somatic embryos through selecting appropriate explant, optimizing the culture media and environmental conditions^9^.

Chinese fir (*Cunninghamia lanceolata* (Lamb.) Hook) is the most important conifer tree species in south subtropical areas of China; its natural distribution extends from 19°30′N to 34°03′N and from 101°30′E to 121°53′E, covering 17 administrative provinces^10^. The Chinese fir plantation area is approximately 9 million ha, accounting for 30% of the national afforestation area, which ensures that it occupies an important position in the forest resources of China^11^. Chinese fir is also a valued timber species because of its rapid growth and high-quality wood, which is used extensively for pulp, paper, and lumber^12^.

Since the 1960s, systematic genetic improvements have been carried out in Chinese fir. Biotechnological approaches such as cloning production^13^, breeding^14^, use of molecular markers^10^, application of tissue culture for propagation^15^, and genetic engineering^16^ have all been employed to develop and produce Chinese fir trees with better-quality wood. Conventional breeding of Chinese fir is difficult and time consuming due to its long generation time, large genome, and the highly heterozygous genetic background. Despite the high potential of genetic engineering, this technology remains costly, difficult, or impossible in most Chinese firs, mainly due to the absence of an effective *in vitro* regeneration system. Vegetative propagation of Chinese fir is also difficult compared with that of other coniferous species, and traditional propagation methods have difficulty meeting the increasing domestic and international demands for this species^12^. Therefore, *in vitro* production systems are urgently needed as a mass propagation method and a tool for future transgenic manipulation of Chinese fir.

Plant regeneration from tissue culture has been reported in Chinese fir using cotyledon and hypocotyl explants derived from greenhouse-grown seedlings^15^, but it has mainly occurred through adventitious bud formation and subsequent shoot and plantlet development. Only a few studies have been reported on SE of Chinese fir. SE has been induced in Chinese fir with the initial cotyledon and hypocotyl explants and mature zygotic embryos by direct SE pathway, i.e., all of the somatic embryos were produced by direct SE without mediation of the embryogenic callus^17, 18^. Although the highest induction proportion was almost 10%, but as for the single explant, the yield of somatic embryos are few, and it is difficult for a Chinese fir definite phenotype to regenerate sufficient somatic embryos in a short time by direct SE pathway. The limited somatic embryos yield not only has difficulty meeting the needs of mass propagation for Chinese fir elite genotypes, but also restricts its application as the tool in Chinese fir genetic improvement. It is necessary to explore the other methods of improving the efficiency of Chinese fir SE. Intermediate callus formation is an indirect SE pathway, and a lot of reports has described somatic embryo formation via an intervening callus such as in *Pinus* species^19^. In addition, the embryogenic callus can be proliferated for many generations and has the potential to develop into somatic embryos if cultured under appropriate conditions. Therefore, the embryogenic callus ensures mass propagation for trees of elite and high-value genotype^20^, and the indirect SE pathway via embryogenic callus intermediation may be another method to enhance Chinese fir SE yield.

Embryogenic callus can be generated from a variety of tissues in coniferous species, including immature and mature zygotic embryos^21^ and vegetative shoot apices^22^. However, unlike flowering plants, in which the embryogenic process is often initiated by mature explants, embryogenic callus in coniferous species is always generated from juvenile tissues, such as immature embryos^23^. Since the first report that morphologically and physiologically mature somatic embryos could produce vigorous plantlets from embryogenic callus induced from immature *Picea abies* embryos^8^, the greatest success in inducing SE in coniferous species has occurred with immature zygotic embryos. However, no reports are available on the induction of somatic embryos from explants of immature Chinese fir zygotic embryos. The main objective of this study was to develop a reliable method for inducing SE in tissues from mature Chinese fir trees with an elite genotype. The embryogenic potential of three developmental stages of immature embryos was investigated. The ability of plant growth regulators and genotypes to induce an embryogenic response and the effects of ABA and PEG on somatic embryos maturation were also studied.

## Materials and Methods

### Plant materials

**Figure 1:**
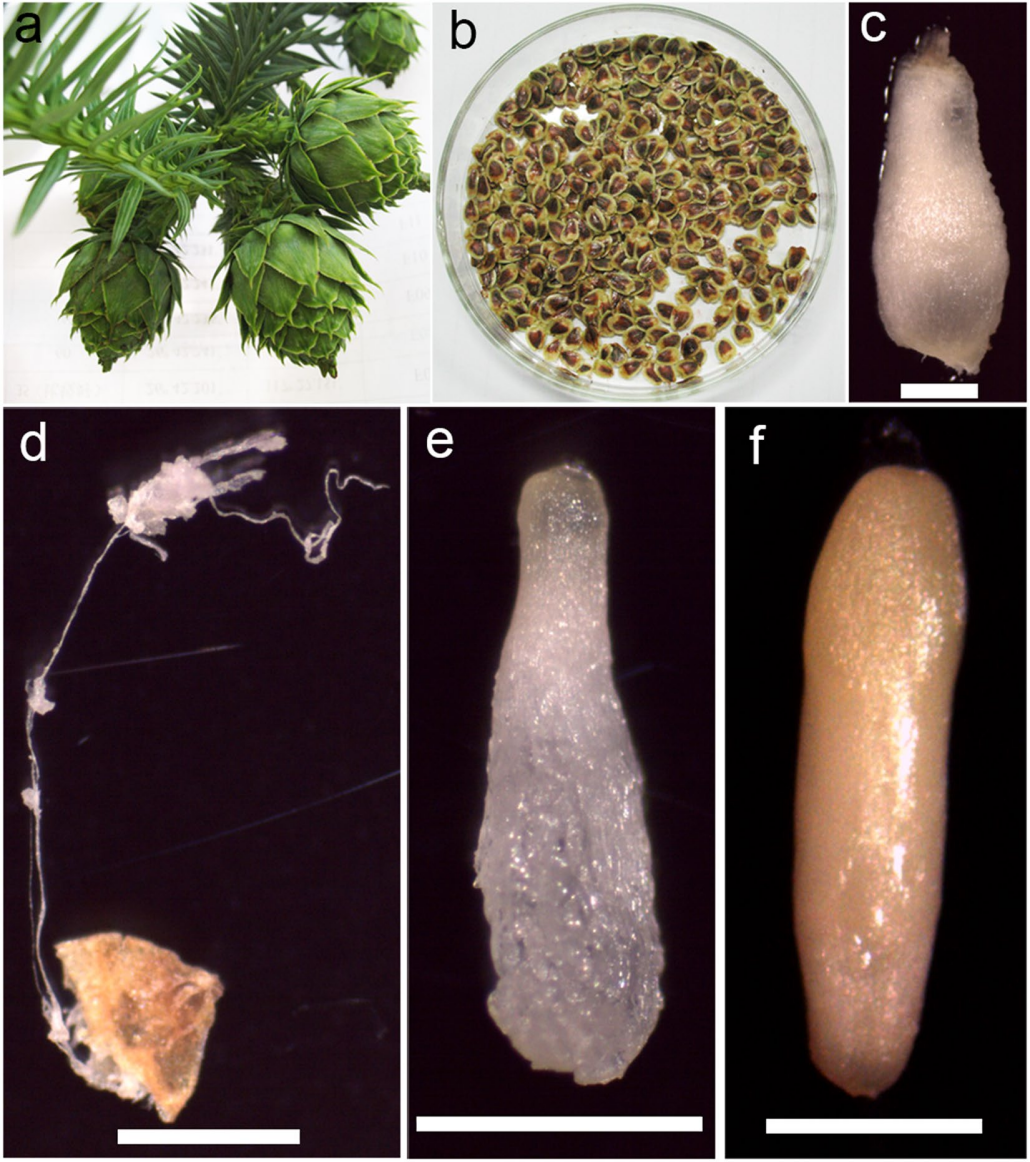
Developmental stages of *Cunninghamia lanceolate* zygotic embryos collected on different dates and from different seed orchards. (**a**) Green female cones. (**b**) Immature seeds. (**c**) Megagametophyte. (**d**) Embryos at the cleavage polyembryony stage. (**e**) Embryos at the dominant embryo stage. (**f**) Embryos at the precotyledon stage. *Bars* 1 mm.

Green female cones (Fig. 1a) enclosing immature seeds (Fig. 1b) were collected from 25 genotype open-pollinated (OP) trees growing in three Chinese fir clonal seed orchards of southern China. Seven OP trees (named F1–F7) were from the Jiangle seed orchard (latitude: 26°42′12″N, longitude: 117°27′11″W) and were sampled on 7, 17, and 27 July 2013. Ten and eight OP trees (G1–G10 and H1–H8, respectively) were from the Xiaokeng (latitude: 24°41′51″N, longitude: 113°49′01″W) and Kaihua (latitude: 29°08′52″N, longitude: 118°24′55″W) seed orchards, respectively, and were sampled on 17 and 27 July and 8 August 2013. About 20 cones of each genotype tree were collected at each sampling time, taken to the laboratory immediately with an incubator for the subsequent experiment. The temperature inside the incubator during transport was 4–10 °C.

Immature seeds were extracted from the dissected cones and disinfected by immersion in 70% (w/v) ethanol for 1 min, and then in 0.1% (w/v) mercuric chloride solution for 10 min, followed by three rinses in sterile distilled water under a laminar flow hood. After seed coats were removed, the immature zygotic embryos were stripped out aseptically from megagametophytes with scalpel and tweezers. They were used as the initial explants and placed horizontally onto a disposable sterile Petri dish (diameter, 60 mm) with medium for the subsequent embryogenic tissue induction culture. Based on the fact that cleavage polyembryony-stage embryos were tiny and vulnerably dehydrated, it was not suitable to directly squeeze them out from the megagametophytes, so the total megagametophytes enclosing cleavage polyembryony-stage embryos were used as the initial explants. While the other developmental stages of embryos, the initial explants were also immature zygotic embryos.

### Developmental stages of the zygotic embryos

The developmental stages of the zygotic embryos were evaluated at each collection date and for each mother tree. Ten randomly chosen seeds were dissected with a scalpel and tweezers, and the embryos were examined under a stereomicroscope.

### Embryogenic tissue induction and proliferation

The initial explants from all collection dates including immature zygotic embryos and megagametophytes were cultured on callus induction medium (Table 1), i.e., DCR basal medium^24^ supplemented with 30 g L^−1^ sucrose, 0.5 g L^−1^ casein hydrolysate, 0.4 g L^−1^ glutamine, 5 g L^−1^ phytagel, 1.0 g L^−1^ activated carbon, and plant growth regulators. Compared with one another, the media CM1–CM7 and CM8–CM11 are characterized by higher concentrations of auxin analog (i.e., 2,4-D) and cytokinin analogs (i.e., BA and TDZ), respectively, and they were used to determine the effects of different plant growth regulators on embryogenic tissue induction. All culture media were brought to pH 5.8 before autoclaving at 121 °C for 20 min. 30–50 explants for each genotype OP tree and medium were placed on three 60 cm diametric petri dishes containing sterile induction medium. Three replicates were used per each induction treatment. The explants were incubated in medium for 6 weeks in the dark at 25 ± 1 °C without subcultures before being transferred onto proliferation medium. After a 6-week culture, the numbers of embryos producing embryogenic and non-embryogenic calluses were observed, and the rates of embryogenic and non-embryogenic calluses induction were calculated. The genotypes of the embryogenic calluses derived from each individual seed were recorded.

**Table 1:** Embryogenic tissue induction media (CM1–CM11).

Growth regulators	Callus induction medium^a^ (mg L^−1^)										
	CM1	CM2	CM3	CM4	CM5	CM6	CM7	CM8	CM9	CM10	CM11
2,4-D	1.0	1.0	2.0	2.0	0.5	1.5	1.5	—	—	—	0.2
BA	0.2	0.5	0.2	0.5	0.2	—	0.5	1.0	1.5	2.0	1.5
KN	—	0.3	0.3	—	—	0.3	0.3	—	—	—	—
NAA	—	—	—	—	—	—	—	0.2	0.2	0.2	
TDZ	—	—	—	—	—	—	—	0.004	0.004	0.004	0.004

^a^Each medium was composed of DCR basal medium and 30 g L^−1^ sucrose, 0.5 g L^−1^ casein hydrolysate, 0.4 g L^−1^ glutamine, 5 g L^−1^ phytagel, 1.0 g L^−1^ activated carbon, and plant growth regulators.

After a 6-week induction, the cultures were grown on DCR proliferation medium supplemented with the same quantities of sucrose, casein hydrolysate, glutamine, phytagel and activated carbon. The PM1 and PM2 proliferation media containing different plant growth regulators were used for subsequent cultures. PM1, containing 0.2 mg L^−1^ 2,4-D and 0.1 mg L^−1^ KN, and PM2, which was the same as CM9, were used for proliferation of cultures from CM1–CM7 and CM9–CM11, respectively. The cultures were subdivided, transferred onto proliferation media PM1 and PM2, and subcultured biweekly. Three successive transfer cultures later, the proliferation culture effects were assessed under an optical microscope and a stereomicroscope.

### Somatic embryos induction

In order to detect the combinatory effect of different plant growth regulators on SE, embryos collected from G1–G10 on the first sampling date were incubated in somatic embryo induction media SEI1-SEI5 (Table 2). Media SEI1-SEI5 were also DCR basal medium supplemented with the same quantities of sucrose, casein hydrolysate, glutamine, phytagel, and activated carbon as induction medium. The plant growth regulator content of media SEI1–SEI5 was as shown in Table 2. Among these five media, the second medium was the same concentration of plant growth regulators as CM9 and PM2, and renamed as SEI2. About 30 embryos were placed on each somatic embryo induction medium. The embryos were subcultured biweekly. After 6 successive transfer cultures in the dark at 25 ± 1 °C, the numbers of embryos producing SE were observed, and the rates of somatic embryo induction were calculated. Three replicates were used per each induction treatment.

**Table 2:** Effect of plant growth regulators combination on somatic embryogenesis.

Somatic embryo induction medium^a^	Plant growth regulators (mg L^−1^)					Response (%)		
	2,4-D	NAA	6-BA	KN	TDZ	Buding	SE	Callus
SEI1	0.5	—	—	0.5	—	0	2.62 ± 0.21	97.38 ± 0.21
SEI2	—	0.2	1.5	—	0.004	40.04 ± 0.92	8.45 ± 0.66	51.51 ± 0.79
SEI3	1.5	—	—	0.2	—	0	0	100
SEI4	1.5	—	0.2	—	—	0	0	100
SEI5	1.5	—	0.2	0.2	0.004	0	0	100

^a^Each somatic embryo induction medium was composed of DCR basal medium and 30 g L^−1^ sucrose, 0.5 g L^−1^ casein hydrolysate, 0.4 g L^−1^ glutamine, 5 g L^−1^ phytagel, 1.0 g L^−1^ activated carbon, and plant growth regulators.

### Maturation of somatic embryos

Maturation experiments were carried out with the H6-6# embryogenic cell line, which was derived from mother tree H6. Embryogenic tissue masses were transferred to DCR medium supplemented with various concentrations of ABA and PEG, 30 g L^−1^ sucrose, 0.5 g L^−1^ casein hydrolysate, 0.4 g L^−1^ glutamine, 2.0 g L^−1^ activated carbon, and 5 g L^−1^ phytagel to optimize the maturation conditions (Table 3). Four concentrations of ABA (25, 50, 75, and 100 μmol L^−1^) were added to DCR medium to determine the effects of ABA on somatic embryo maturation, these four ABA treatment media were named M1-M4 respectively. Three concentrations of PEG6000 (50, 100, and 150 g L^−1^) were used in the maturation medium to investigate the effects of osmotic pressure, these three PEG treatment media were named M5–M7 respectively. Medium M0 was control, namely, this medium was free of ABA and PEG whereas supplemented with same concentration of sucrose, casein hydrolysate, glutamine, activated carbon, and phytagel. Eight embryogenic tissue masses, approximately 0.2 g (fresh weight) each, were transferred to ABA- and PEG-containing media with 10 replicates each. The cultures were maintained at 25 ± 1 °C in the dark. After a 2-week pre-culture, the numbers of early somatic embryos from the two kinds of pre-maturation media were scored in three randomly chosen tissue masses under a stereomicroscope, and the optimal ABA and PEG concentrations were determined. Based on the statistical results of early somatic embryos under different concentrations of ABA and PEG, embryogenic tissue masses were then incubated in DCR maturation medium supplemented with 50 μmol L^−1^ ABA and 100 g L^−1^ PEG. The tissues were subcultured every 4 weeks. After an 8-week culture, the yield and morphology of the somatic embryos were recorded in three replicates.

**Table 3:** Effect of ABA and PEG on somatic embryo maturation.

Maturation medium^a^	ABA content (μM)	PEG6000 content (g L^−1^)	Early somatic embryos yield^b^
ME0	0	0	0.00a
ME1	25	0	20.33 ± 0.98b
ME2	50	0	35.67 ± 1.19c
ME3	75	0	16.67 ± 1.66d
ME4	100	0	9.33 ± 0.72e
ME5	0	50	0.00a
ME6	0	100	5.00 ± 0.94f
ME7	0	150	0.00a
ME8	50	100	53.33 ± 2.88g

^a^Maturation media ME1–ME8 were composed of DCR basal medium supplemented with various concentrations of ABA and PEG, 30 g L^−1^ sucrose, 0.5 g L^−1^ casein hydrolysate, 0.4 g L^−1^ glutamine, 2.0 g L^−1^ activated carbon, and 5 g L^−1^ phytagel.

^b^Duncan's new multiple range test was used to detected differences at P < 0.05. Different letters within a column indicate a significant difference.

### Statistical analysis

Analysis of variance was used to detect differences. All data analyses were performed using the SPSS v. 17.0 statistical software package (SPSS, Inc., Chicago, IL, USA). P-values ≤ 0.05 were considered significant.

## Results

### Induction of embryogenic tissue

**Figure 2:**
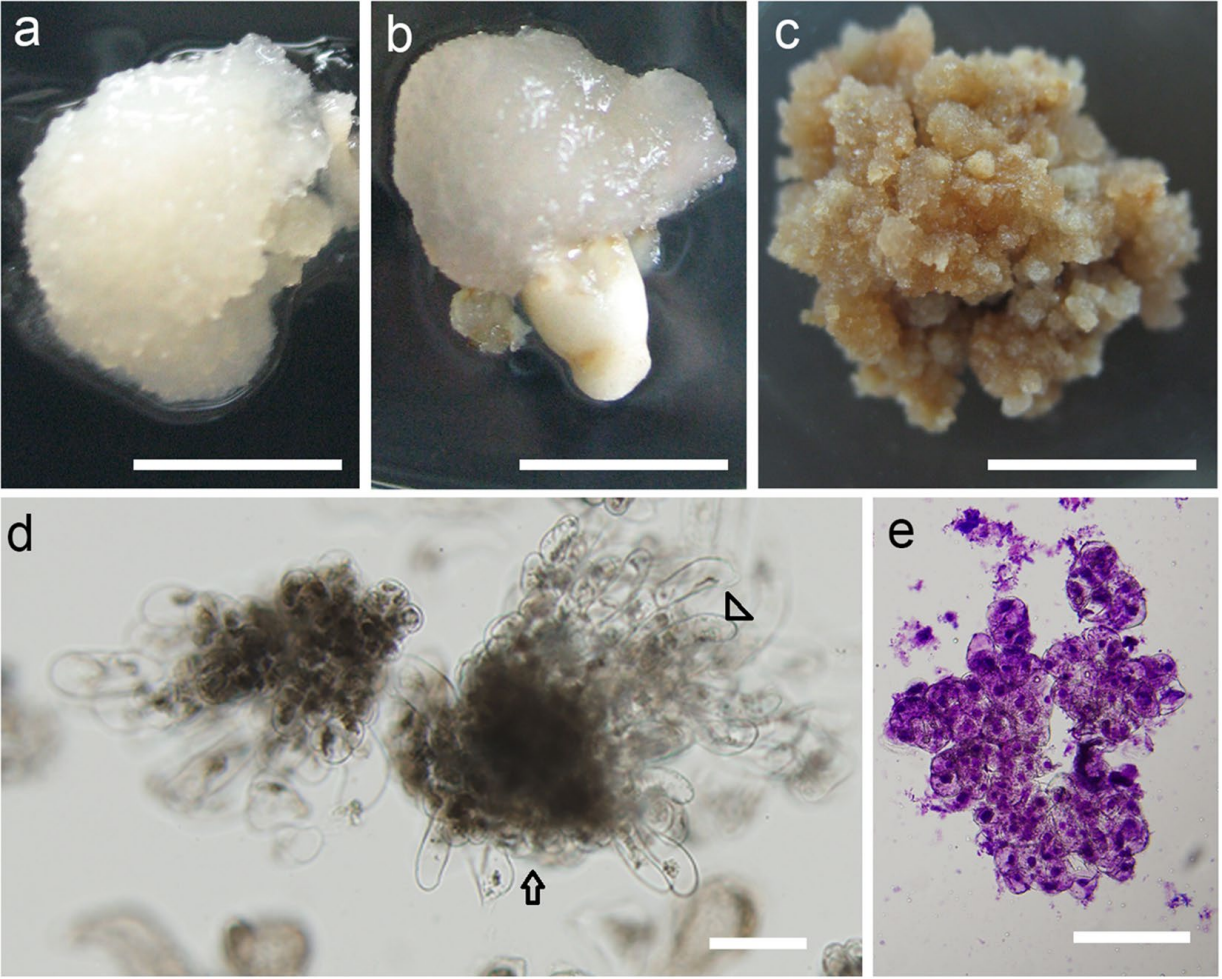
Morphological aspects of *Cunninghamia lanceolate* embryogenic cultures. (**a**) Embryogenic callus formed from an immature embryo. (**b**) Embryogenic callus formed from a megagametophyte. (**c**) Non-embryogenic callus. (**d**) Polarized embryogenic suspensor masses. Arrow shows embryogenic cells. Arrowhead shows suspensor cells. (**e**) Non-embryogenic callus cells stained with fuchsine. (**a–c**) *Bars* 5 mm, (**d**,**e**) *bars* 100 μm.

The immature embryos became bulgy and loose and were white in color after 7 days of incubation in induction media CM1–CM7. A fraction of the embryos gradually developed a brown color, demonstrating symptoms of necrosis. After 6 weeks, the surviving immature embryos developed into two kinds of calluses by dedifferentiation; the calluses were different colors and textures. Most of the white or pale-yellow, crystal and soft calluses (Fig. 2a) were embryogenic calluses characterized by numerous bipolar structure embryos formed by two different cell types, i.e., small meristematic cells and elongated suspensor cells (Fig. 2d). Small meristematic cells were densely cytoplasmic and composed the embryonic head, whereas elongated cells with large vacuoles formed the suspensor apparatus. The yellow or yellowish-brown friable or compact calluses (Fig. 2c) were recalcitrant and were rarely embryogenic calluses. The cells of the non-embryogenic calluses were of a similar size but had no cytoplasmic cell clusters. Although they proliferated, no bipolarized structure was observed under the microscope (Fig. 2e). As the culture progressed, these calluses turned dark brown, disaggregated, and became necrotic. The other explant type from the intact megagametophyte could also be used to induce an embryogenic callus. As shown in Fig. 2b, the callus ball was ejected from micropyle, and the morphological characteristics of the callus were very close to those of the embryogenic callus induced by immature embryos. Microscopic observations revealed that these calluses were also embryogenic calluses.

Immature embryos showed similar phenotypic changes, such as bulging, in the initial 7 days of incubation in induction media CM8–CM11. As the culture progressed, some of the embryos continued to swell until to approximately 2 times bigger than the initial embryos; some of the embryos gradually became loose and then partly or completely dedifferentiated into callus 6 weeks later. It was difficult to distinguish which kind of cultures, such as swelled embryos, partly and completely dedifferentiated calluses, were embryogenic, so all of the viable cultures including embryos and calluses were recorded and calculated as surviving rate.

### Effects of immature embryo developmental stage on the embryogenic response

The microscopic morphological observations indicated that the immature embryos collected from 25 OP trees at three sampling times could be divided into three development stages: cleavage polyembryony (Fig. 1d), dominant embryo (Fig. 1e), and precotyledon (Fig. 1f). Immature embryos from F1–F7 and H1–H8 collected during the first sampling time were in the cleavage polyembryony stage, and those collected during the second and third sampling times were dominant embryo and precotyledon stages, respectively. Immature embryos sampled from mother trees G1–G10 during the first sampling time were in the dominant embryo stage, and those collected during the second and third sampling times were in the precotyledon stage.

**Figure 3:**
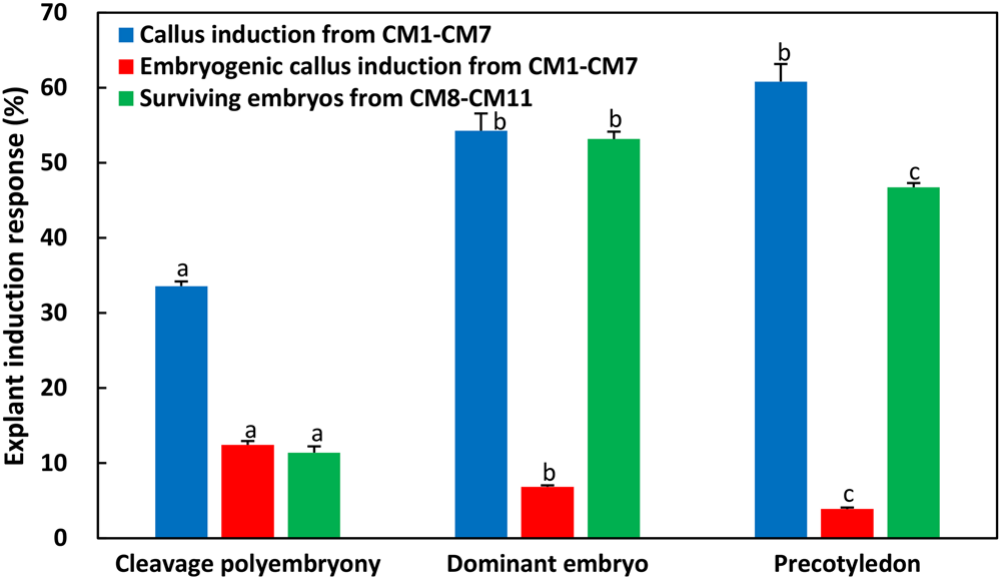
Effects of the three embryo developmental stages on embryogenic tissue induction. Cleavage polyembryony-, dominant-, and precotyledon-stage embryos were cultured in embryogenic tissue induction media CM1–CM11 for 6 weeks, after which, the rates of callus (represented by blue columns) and ESM (represented by red columns) induction from CM1–CM7 and the survival rate of embryos (represented by green columns) from CM8–CM11 were determined. Duncan's new multiple range test was used to detect differences at P < 0.05.

These three developmental stages of immature embryos presented significant differences in embryogenic responses after 6 weeks of dedifferentiation culture on induction medium (Fig. 3). As shown in Fig. 3, the average rate of callus (including embryogenic and non-embryogenic callus) induction on media CM1–CM7 increased gradually along with embryo development, ranging from 33.58% cleavage polyembryony to 54.27% dominant embryos and 60.82% precotyledons. Among all the induced tissues, only a small proportion were embryogenic calluses; and the frequency with which explants developed into embryogenic calluses was also significantly affected by embryo developmental stage (*P* ≤ 0.05). In contrast to the callus, the induction rate of embryogenic calluses decreased gradually, as 12.44% cleavage polyembryony-stage embryos and 6.86% dominant embryos produced embryogenic calluses, both higher than the 3.89% of precotyledon embryos.

Only 11.4% of cleavage polyembryony embryos survived; the remainder turned brown and lost viability during culture in induction media CM8–CM11. Although these embryos survived the primary induction culture, the explants were recalcitrant to embryogenesis and dedifferentiated into embryogenic callus or regenerated adventitious buds in subsequent proliferation cultivation. Only those in the dominant embryo stage gradually became bulgy and looked healthier than the precotyledon and cleavage polyembryony embryos. This dominant embryo stage had a 53.17% survival rate, which was higher than the 46.74% rate of the precotyledon embryos.

### Effects of auxin/cytokinin concentrations on the embryogenic response rate

**Figure 4:**
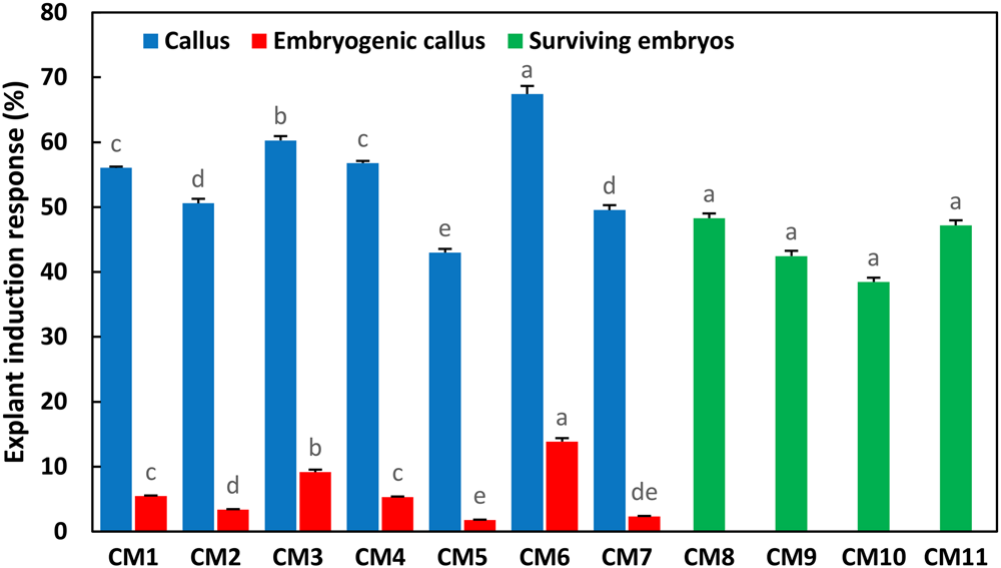
Effects of induction media CM1–CM11 on embryogenic tissue induction. Immature embryos from 25 OP trees in three developmental stages were cultured for 6 weeks in embryogenic tissue induction media CM1–CM11, and the average frequency of callus (represented by blue columns) and embryogenic callus (represented by red columns) induction from CM1–CM7 and the survival rates of embryos (represented by green columns) from CM8–CM11 were determined. Duncan's new multiple range test was used to detect difference at P < 0.05.

**Figure 5:**
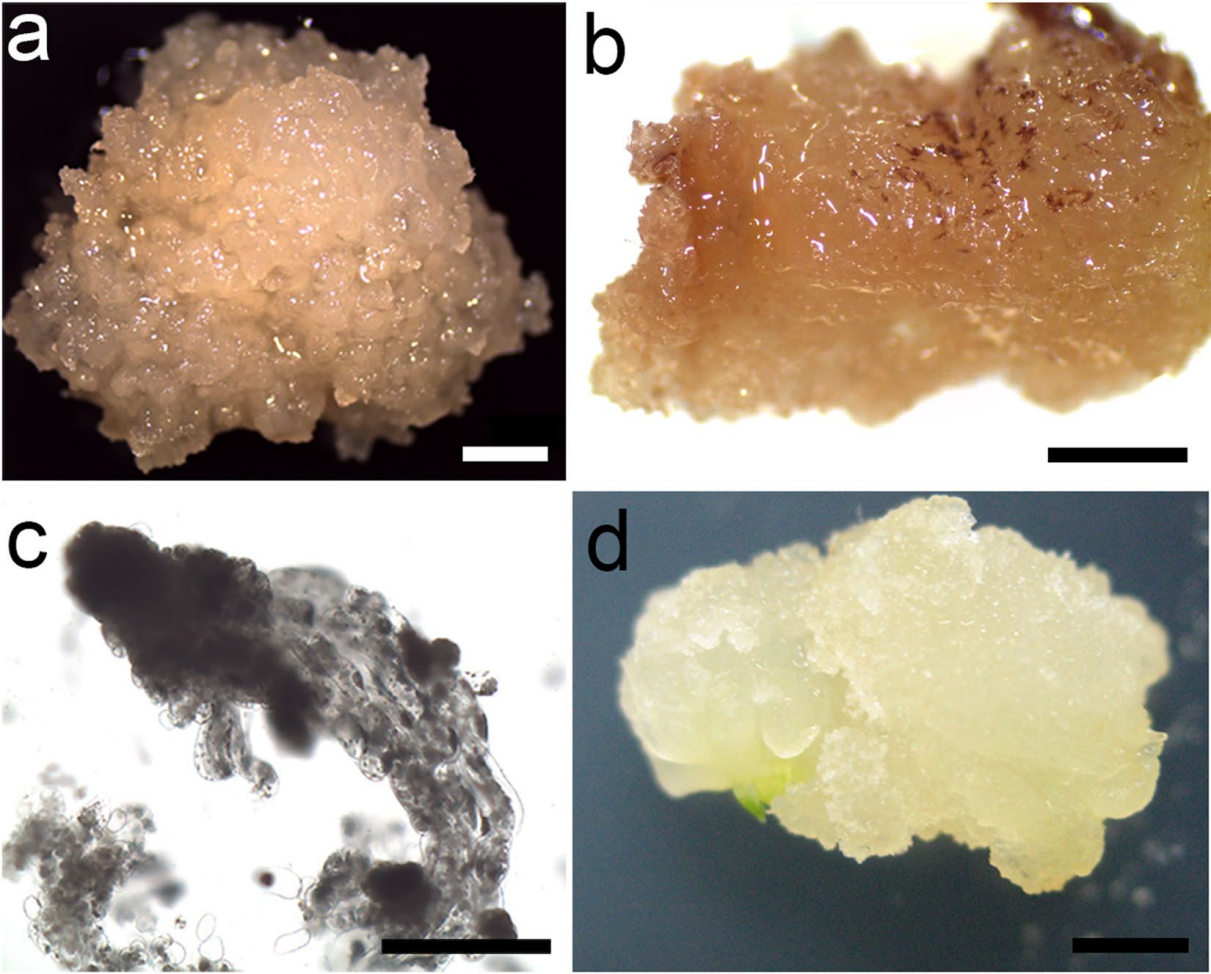
Effects of proliferation media PM1 and PM2 on cultures proliferation. (**a**) Further development of embryogenic callus in proliferation medium PM1. (**b**) Cultures mainly induced by cleavage polyembryony embryos dedifferentiated into soft and easy browning callus in proliferation medium PM2. (**c**) Embryogenic suspensor mass structures of the embryogenic callus observed under a microscope; these structures consisted of clusters of small meristematic cells with dense cytoplasm forming emerging embryonal heads with attached elongated suspensor cells. (**d**) Cultures mainly induced by precotyledon embryos dedifferentiated into hard and difficult to proliferate callus in medium PM2. (**a**,**b**,**d**) *bars* 1 mm, (**c**) *bar* 100 μm.

Two types of induction media with auxin and cytokinin were tested to determine how they affected immature embryos. Media CM1–CM7 were characterized by higher auxin concentrations (0.5–2.0 mg L^−1^ 2,4-D), which were 1.25–5 times greater than the cytokinin concentrations (BA and KN) (Table 1). In these seven induction media, 42.99–67.42% of the initial explants responded to auxin and cytokinin and formed calluses, and 1.78–13.86% of the immature embryos dedifferentiated into embryogenic calluses (Fig. 4). The largest frequency of callus (67.42%) and embryogenic callus (13.86%) inductions were observed in medium CM6 supplemented with 1.5 mg L^−1^ 2,4-D and 0.3 mg L^−1^ KN, i.e., five-fold difference in the auxin/cytokinin rate. As the total concentration and proportions of auxin and cytokinin decreased, the rates of callus and embryogenic callus induction decreased gradually. The minimum was 42.99% and 1.78%, respectively, appearing in medium CM5 supplemented with 0.5 mg L^−1^ 2,4-D and 0.2 mg L^−1^ BA. After a 6-week induction, the calluses from media CM1–CM7 were transferred into proliferation medium PM1. Non-embryogenic calluses began to turn dark brown and became necrotic after 6 weeks of proliferation culture. The embryogenic callus appeared to be a small nodular structure on the callus surface (Fig. 5a). These structures were white or pale yellow, shiny, and soft (Fig. 5a). Microscopic observations revealed that these structures further developed into embryogenic suspensor masses consisting of clusters of small meristematic cells, with dense cytoplasm forming emerging embryonal heads and elongated suspensor cells compared with the initial calluses that formed in induction medium (Fig. 5c).

Media CM8–CM11 characterized by higher cytokines concentrations were supplemented with different concentrations of BA (1.0–2.0 mg L^−1^), 0.2 mg L^−1^ NAA or 2,4-D, and 0.004 mg L^−1^ TDZ. After 6 weeks of incubation in these media, 38.46–48.29% of the immature embryos survived after primary cultivation (Fig. 4). Although no difference was observed in survival rate, embryos cultured in CM9 were healthier than those in the three other induction media. Based on these results, we chose CM9 as the subsequent proliferation medium, supplemented with the same concentrations of plant growth regulators, and called it PM2. After 6 weeks of induction culture, all surviving embryos were transferred to proliferation medium PM2. During in the subsequent three transfer cultivation, most of the cultures would gradually dedifferentiate into two kinds of calluses, one was soft and easy browning mainly induced by cleavage polyembryony embryos (Fig. 5b), the other was hard and difficult to proliferate mainly induced by precotyledon embryos (Fig. 5d). Only the cultures induced by dominant embryos regenerated adventitious buds or developed into somatic embryos in medium PM2.

### Effect of different plant growth regulators combination on somatic embryogenesis

After 12 weeks of incubation in somatic embryo induction media SEI1–SEI5, embryos collected from G1–G10 on the first sampling date showed significantly different responses for plant growth regulators combination (Table 2).

**Figure 6:**
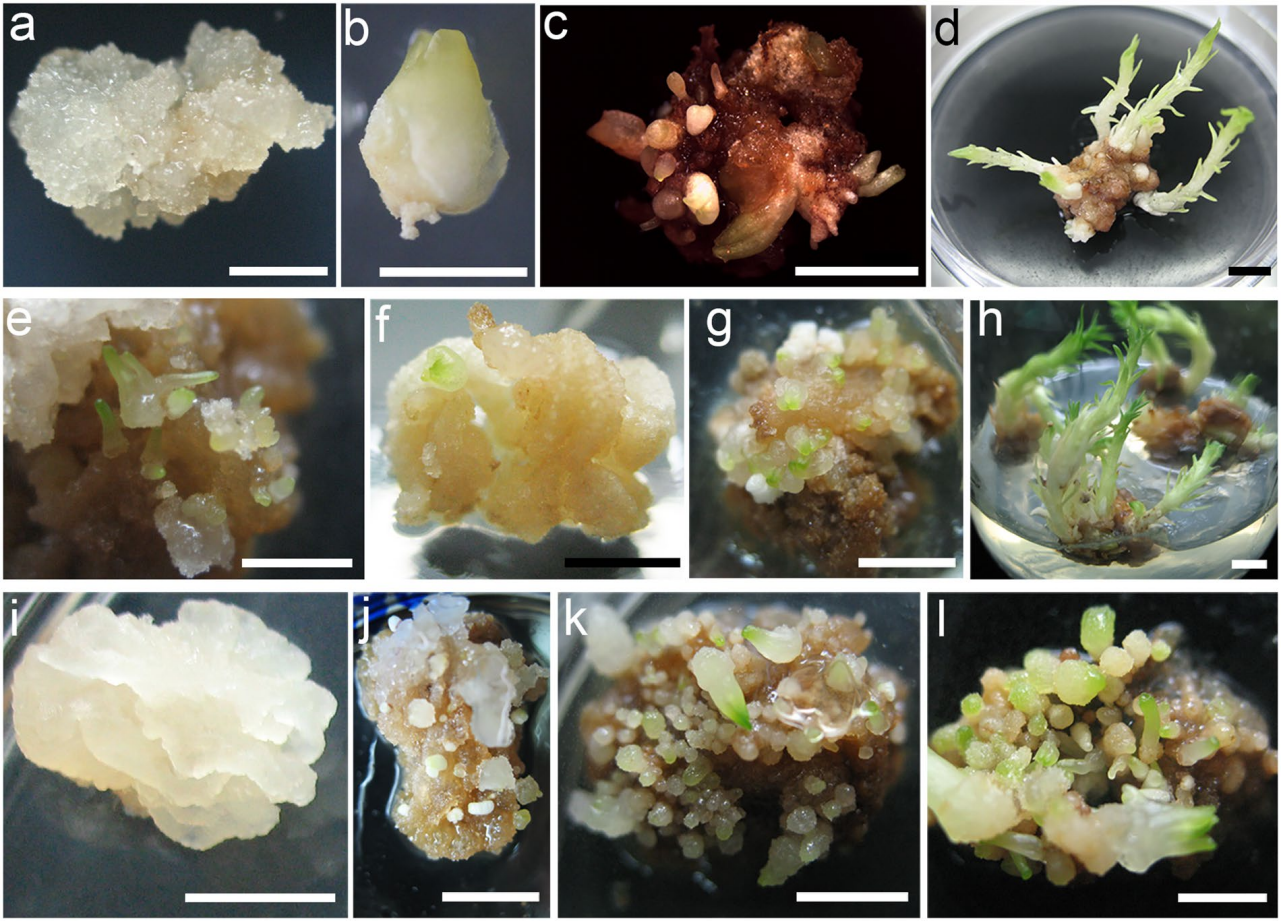
Effect of different plant growth regulators combination on somatic embryogenesis. (**a**) Callus induced by somatic embryo induction medium SEI1. (**b**) Swollen embryos in somatic embryo induction medium SEI2. (**c**) Cup-shaped or somewhat curved-shaped initial buds emerged from the surface of swollen embryos in medium SEI2. (**d**) Elongating adventitious buds in subsequent cultures. (**e**) White cap-shaped or columnar pro-embryos arose from a disaggregated watery callus in SEI1 medium. (**f**) Partly dedifferentiated callus. (**g,h**) Growing adventitious buds from a partly dedifferentiated callus in medium SEI2. (**i**) Completely dedifferentiated callus characterized by white translucent gullies and bulges attached to the surface. (**j–l**) White cap-shaped or columnar pro-embryos arose from a disaggregated watery callus in SEI2 medium. Hundreds of pro-embryos were observed during further proliferation and laurel-green cotyledons appeared from the embryonal head. *Bars* 5 mm.

In somatic embryo induction medium SEI1 supplemented with 0.5 mg L^−1^ 2,4-D and 0.5 mg L^−1^ KN, 97.38% of the survived embryos gradually dedifferentiated into callus during in the initial 4 weeks cultivation. These completely dedifferentiated calluses proliferated but could not developed into somatic embryos in subsequent 8 weeks culture (Fig. 6a). Only 2.62% of the survived embryos not only dedifferentiated into callus but also developed into some cap-shaped or columnar pro-embryos from the surface of callus (Fig. 6e).

After 6 successive transfer cultures in SEI2 containing 0.2 mg L^−1^ NAA, 1.5 mg L^−1^ 6-BA, and 0.004 mg L^−1^ TDZ, 51.51% of the embryos dedifferentiated into callus, and the other embryos regenerated adventitious buds or developed into somatic embryos under the following three conditions. (1) Some of the embryos directly regenerated adventitious buds without undergoing dedifferentiation into a callus. These embryos initially presented swollen hypocotyls in the first 4 weeks (Fig. 6b). Then, initial cup-shaped or somewhat curved-shaped buds emerged from the surface of the swollen embryos during in the next 4 weeks (Fig. 6c). In subsequent subcultures, the buds elongated, and needles came out from the stem (Fig. 6d). (2) Some embryos induced adventitious buds through dedifferentiation into a callus. These embryos gradually proceeded to dedifferentiate in the first 4 weeks (Fig. 6f). During in the next 4 weeks, these embryos generated calluses originating from further dedifferentiation, and adventitious buds sprouted from the calluses (Fig. 6g,h). Embryos regenerating adventitious buds with and without undergoing dedifferentiation into a callus totally accounted for about 40.04% on average (Table 2). (3) On average of 8.45% (Table 2) embryos developed into early somatic embryos through a completely dedifferentiated callus characterized by white translucent gullies and bulges on the surface (Fig. 6i). During subsequent cultivation, the white, cap-shaped or columnar pro-embryos arose from a disaggregated watery callus (Fig. 6j). Hundreds of pro-embryos were observed during further proliferation and were easily separated from the surrounding callus (Fig. 6k). Laurel-green cotyledons appeared from the embryonal head in the next subculture (Fig. 6l).

Somatic embryo induction media SEI3–SEI5 were characterized by high concentration of auxin, i.e., 1.5 mg L^−1^ 2, 4-D (Table 2). No matter sole or combinational employment of cytokines (6-BA, KN andTDZ) in these media, all of the embryos eventually developed into callus in the first 4 weeks. During in the subsequent 8 weeks, these calluses were inflexible and would not regenerate adventitious buds or developed into somatic embryos.

### Effect of mother tree genotype on embryogenic callus induction and somatic embryogenesis

**Figure 7:**
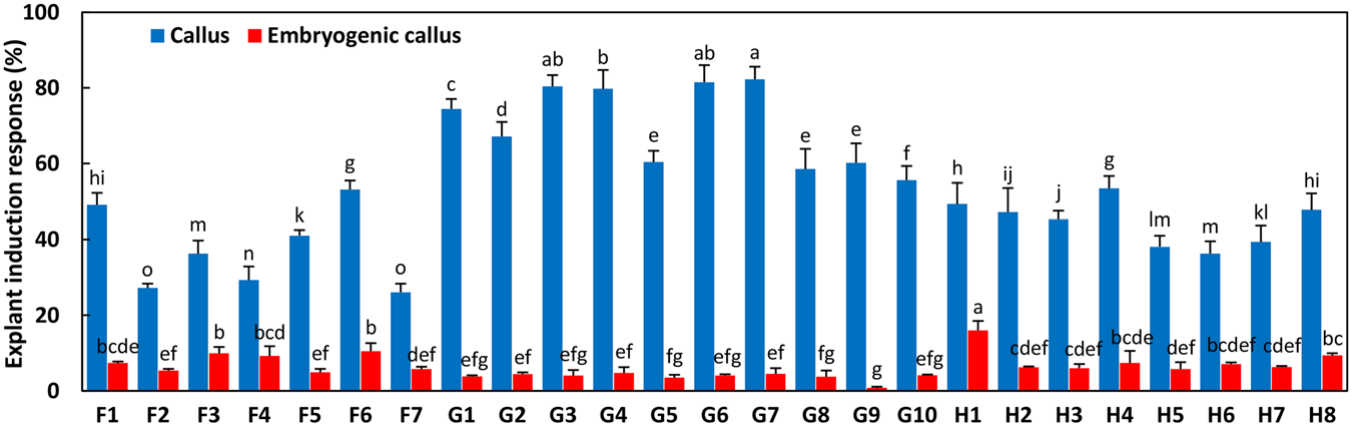
Effect of mother tree genotype on callus and embryogenic callus induction. Duncan's new multiple range test was used to detect differences at P < 0.05.

The frequencies of callus induction and embryogenic callus were significantly affected by the mother tree genotype. As shown in Fig. 7, G7 was the most responsive genotype for callus induction, with the frequency reaching 82.27%, but the rate of embryogenic callus induction was only 4.51%. Although H1 was not the most responsive genotype for callus induction, the rate of embryogenic callus induction reached 16.02%, which was the highest of all 25 mother tree genotypes. In general, genotypes G1–G10, with an average induction frequency of 70.03%, were most suitable for callus induction, which was far higher than the 37.46% found for F1–F7 and 44.60% for H1–H8. About 7.6% and 8% of the immature embryos from F1–F7 and H1–H8, respectively, dedifferentiated into embryogenic callus; this was significantly higher than the frequency in G1–G10 (3.79%).

**Figure 8:**
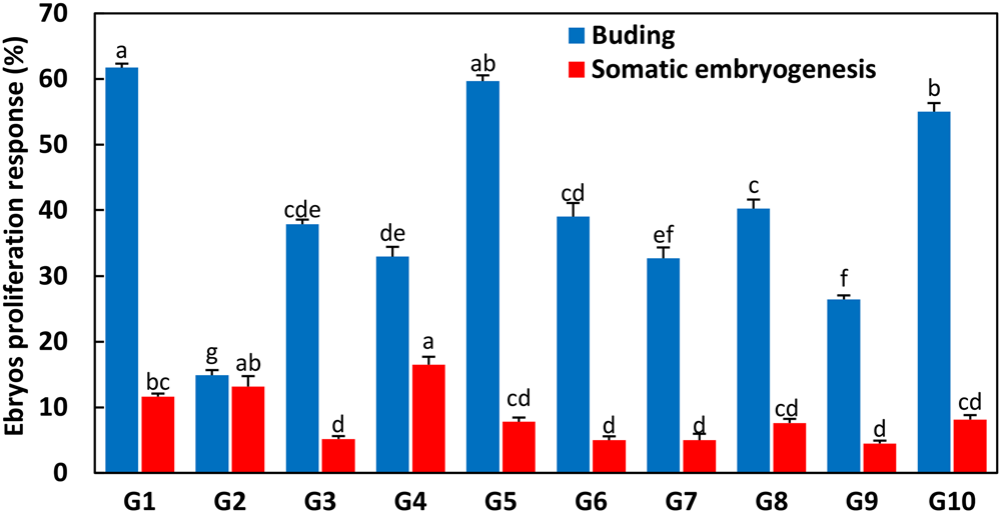
Effect of mother tree genotype on the frequency of adventitious bud formation and somatic embryogenesis. Duncan's new multiple range test was used to detect differences at P < 0.05.

The genotypes of the mother trees also significantly affected the frequency of immature embryos’ developing into adventitious buds and SE. After three successive transfer cultures on proliferation medium PM2, only the surviving embryos collected from G1–G10 on the first sampling date regenerated adventitious buds or developed into somatic embryos. As shown in Fig. 8, G1 had the highest adventitious bud induction rate, 61.72%, whereas G2 had the smallest frequency, 14.9%. The most responsive genotype in SE was G4, in which the frequency reached 16.51%, whereas the lowest rate of SE among G1–G10 was observed in G9 (4.49%).

### Effect of ABA and PEG on maturation of somatic embryos

**Figure 9:**
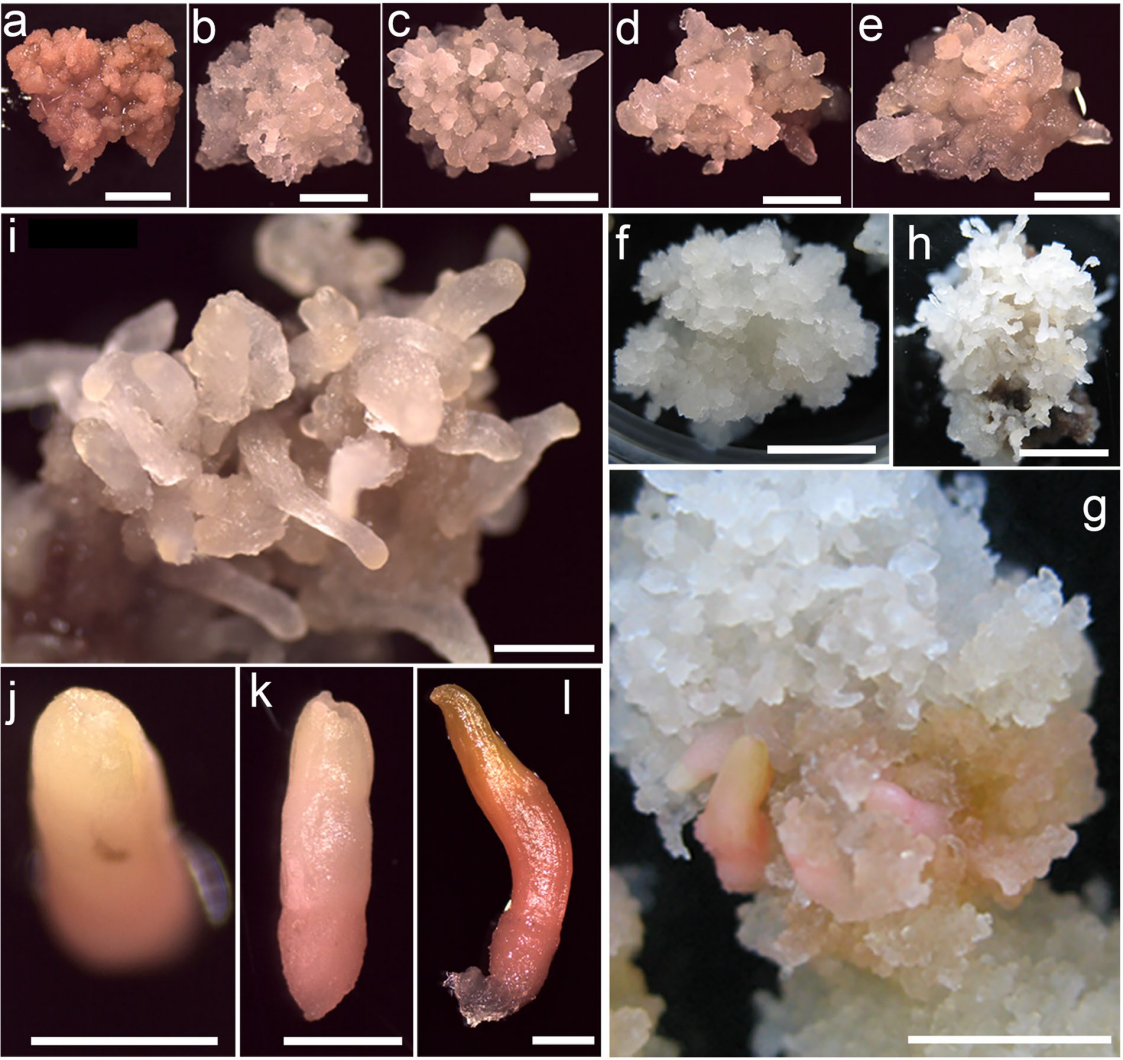
Effects of ABA and PEG on the potential for somatic embryogenesis. (**a–e**) Early somatic embryo developmental potential after 2 weeks’ cultivation of embryogenic calluses in maturation media supplemented with five concentrations of ABA (0, 25, 50, 75, and 100 μmol L^−1^) respectively. (**f–h**) Early somatic embryo developmental potential in maturation media supplemented with three concentrations of PEG6000 (50, 100, and 150 g L^−1^), respectively. (**i**) Early somatic embryo developmental potential in maturation media supplemented with 50 μmol L^−1^ ABA and 100 and 150 g L^−1^ PEG6000. (**j–l**) As maturation progressed, early somatic embryos (**j**) gradually differentiated into early cotyledonary embryos (**k**) and finally developed into mature embryos (l). (**a–h**) *bars* 5 mm, (**i–l**) *bars* 1 mm.

Transferring the proliferating culture to maturation medium without auxins or cytokinins significantly affected the growth of embryogenic tissue and the development of somatic embryos. Embryogenic tissue incubated in control medium ME0 without ABA or PEG grew slowly, and the expansion ability decreased in the first week. Two weeks later, although few early somatic embryos emerged from the surface of embryogenic tissue masses, these embryos were recalcitrant for further development and gradually began to turn dark brown and necrotic, finally, no further developmental somatic embryos were observed in control medium (Fig. 9a). In contrast with control, embryogenic tissue would keep pale-yellow in color and soft in texture when they were incubated in medium including four concentrations of ABA (25, 50, 75, and 100 μmol L^−1^). ABA treatments not only prevented embryogenic tissues from browning but also resulted in rapid growth of somatic embryos. In the first week, lots of translucent bulges emerged from the surface of embryogenic tissue masses. As the culture progressed, these bulges grew quickly and gradually showed the early characteristics of somatic embryos including darker embryonal heads and lighter elongated suspensors (Fig. 9b–e). These early somatic embryos could be apparently isolated and identified after 2 weeks cultivation in maturation media supplemented with 25–100 μM ABA. Furthermore, different concentration of ABA significantly affected the yield of early somatic embryos. The ABA contents, followed (in parentheses) by early somatic embryo yield per approximate 0.2 g (fresh weight) embryogenic tissue mass were 25 μM (20.33), 50 μM (35.67), 75 μM (16.67), and 100 μM (9.33) (Table 3). These results show that ABA promote the growth and development of somatic embryos at relatively low concentrations 25–50 μM, but had a strong suppressive effect at relatively high concentrations 75–100 μM. The number of embryos increased and then decreased as ABA content increased from 25 to 100 μM. The largest numbers of white, cap-shaped or columnar early somatic embryos were generated on maturation medium ME2 containing 50 μM ABA (Fig. 9c).

Different with the browning in control, embryogenic tissues presented white color when they were cultured in maturation medium including 50–150 g L^−1^ PEG6000, which had a similar effect with ABA in preventing embryogenic tissue browning. PEG was also used as an agent of osmotic pressure to adjust the water potential of maturation medium, then create the drought stress condition to accelerate somatic embryos development. In this study Chinese fir watery embryogenic tissues were gradually dehydrated in PEG-containing medium then turned compact, and the higher concentration of PEG the more severe dehydration. 50 g L^−1^ PEG6000 treatment obviously induced plentiful translucent bulges from the surface of embryogenic tissue masses, but these bulges failed to develop into mature somatic embryos in subsequent cultivation (Fig. 9f). When the concentration of PEG600 was up to 150 g L^−1^, the suspensors were so dehydrated that most of them wilted (Fig. 9h); finally the embryogenic tissue did not develop into healthy somatic embryos. Only in medium ME6, which contained 100 g L^−1^ PEG600, we detected an average of five embryos with distinct cotyledons and elongated hypocotyls generated from embryogenic tissue (Table 3, Fig. 9g).

Based on these results, a further experiment was performed with maturation medium ME8 supplemented with the optimal concentrations of ABA (50 μM) and PEG6000 (100 g L^−1^) to evaluate the maturation of embryogenic tissue. The simultaneous use of ABA and PEG in the maturation medium also had a significant restrained effect on embryogenic tissue browning. An average of 53.33 early somatic embryos with a columnar head and elongated suspensors were generated from the surface of embryogenic tissue after 2 weeks of culture (Fig. 9i). The yield of early somatic embryos improved significantly when ABA and PEG6000 were included in the maturation medium compared with those obtained in maturation medium supplemented with only ABA or PEG6000 (Table 3). As the maturation culture progressed, the head region of the early somatic embryos gradually formed the cotyledon, and the suspensor developed into the hypocotyl (Fig. 9j–l).

## Discussion

This is the first report on inducing SE in Chinese fir using explants of immature zygotic embryos derived from mature trees saved in seed orchards as elite and valuable germplasm resources. Previous studies on Chinese fir SE used the initial explants of cotyledons and hypocotyls from 1-week-old seedlings^17^ or mature zygotic embryos from mature trees^18^. Somatic embryos generated from the explants of cotyledons, hypocotyls, and mature zygotic embryos by direct SE pathway when they were cultured in DCR medium supplemented with 1.0 mg L^−1^ BA, 0.002 or 0.003 mg L^−1^ TDZ, and 0.1 mg L^−1^ NAA for 35 days. Only 2% of mature embryos and <10% of cotyledons and hypocotyls developed into somatic embryos, and most of the explants regenerated adventitious buds, indicating that Chinese fir is recalcitrant to regeneration via SE. Subsequently, different developmental stages of immature Chinese fir zygotic embryos have also been used as initial explants to induce SE^25^, but only a few embryogenic calluses were obtained, and no somatic embryos were generated due to unrestrained necrosis once the embryogenic calluses were cultured in maturation media. These results indicate that it is almost impossible to induce somatic embryos indirectly through the embryogenic callus in Chinese fir.

In the present study, we developed two efficient Chinese fir SE systems with initial explants of different developmental stages of immature zygotic embryos. One system was based on cleavage polyembryony-staged embryo due to their higher potential for inducing embryogenic calluses compared with other stages when cultured on DCR medium supplemented with high concentrations of auxin/cytokinin. Somatic embryos were produced from the induced embryogenic callus once they were cultured on maturation medium without plant growth regulators and supplemented with appropriate concentrations of ABA and PEG6000. In contrast, the other system used immature dominant zygotic embryos as the initial explants because of their high embryogenic response when cultured in low concentrations of auxin/cytokinin. The induced embryogenic cultures finally developed into somatic embryos after successive transfers under the same auxin/cytokinin conditions. All of the somatic embryos induced by these two Chinese fir SE systems were through indirect SE pathway. In the first system, on average of 12.44% cleavage polyembryony-stage embryos developed into embryogenic callus; as for the definite induction medium and mother tree genotype, the embryogenic callus induction rate was higher as 13.68% (CM6) and 16.02% (H1) respectively. These induced embryogenic calluses can be proliferated for many generations and also has the potential to develop into somatic embryos. In the other system, 16.51% dominant zygotic embryos of G4 developed into hundreds of somatic embryos via an intervening callus phase. These two indirect SE systems not only ensure mass propagation of Chinese fir elite genotypes but also could be used as the tool for Chinese fir genetic improvement.

These two Chinese fir SE systems were both based on initial explants of immature zygotic embryos. Previous studies have reported that juvenile tissues, such as immature zygotic embryos or intact megagametophytes, are the most suitable explants for inducing embryogenic tissues of coniferous species^23^. For example, embryogenic calluses were initiated on immature and mature zygotic embryos of *Picea abies*, but the embryogenic callus yield was lower when mature embryos are used than when immature embryos were used^8, 26^. Moreover, the low frequency (around 2%) of Chinese fir SE induction when mature zygotic embryos are used^18^ also indicates that relatively mature tissue may not produce somatic embryos. In our study, we chose immature Chinese fir zygotic embryos as the initial explants for inducing the embryogenic callus. The frequency with which explants developed into embryogenic callus was significantly affected by the developmental stage of the immature embryos, as different developmental stages resulted in different embryogenic responses to the same *in vitro* induction conditions. Similar results were reported for *Pinus radiata* ^27^, in which initiation and proliferation decreased to extremely low frequencies before and after reaching these embryo stages; immature zygotic embryos ranging from cleavage polyembryony to the first “bullet” dominant embryo stage were the most responsive to induction of the embryogenic callus. According to our results, the cleavage polyembryony developmental stage was the most responsive when embryos were cultured in media with a high concentration of auxin.

Plant growth regulators play important roles in the initiation, maintenance, and maturation of somatic embryos^28^. Auxin and cytokinins are key plant growth regulators associated with initiation and proliferation of embryogenic tissue in most species studied, but they also inhibit the development of pro-embryogenic masses into somatic embryos^29^. Auxins and cytokinins in the form of artificial plant growth regulators, such as 2,4-D, NAA, BA, and KN, have most often been used to induce embryogenic tissue, e.g., in *Wolffia arrhizal* ^30^, *Musa acuminata* ^31^, *Deschampsia antarctica* ^32^, *Gentiana kurroo* ^33^, and *Eucalyptus globulus* ^6^. Only 1 μM 2,4-D in the induction medium was able to produce white and friable embryogenic calluses with the explants of sugi immature and mature zygotic embryos^21^. Combined use of 2,4-D and BA significantly enhanced induction of *Abies nordmanniana* embryogenic tissue^34^. In addition, the concentrations of the growth regulators also affect the induction of embryogenic tissue. In the present study, we found that the four highest frequencies of embryogenic callus induction were obtained in media containing high concentrations of auxin/cytokinins, and the highest frequency of embryogenic callus (13.86%) induction was obtained from medium CM6 supplemented with 1.5 mg L^−1^ 2,4-D and 0.3 mg L^−1^ KN, i.e., five-fold more auxin/cytokinin. Although medium CM1 (1.0 mg L^−1^ 2,4-D and 0.2 mg L^−1^ BA) also had five-fold more auxin/cytokinin, the total hormone content was less than that in CM6, which may explain why the induction rate decreased significantly in CM1. Many coniferous species produce embryogenic tissue under high concentrations of auxin/cytokinin, such as in *Picea abies* ^8^, *Picea glauca* ^35^, *Larix leptolepis* ^36^, *Pinus kesiya* ^37^, *Pinus bungeana* ^38^, and *Pinus luchuensis* ^39^.

The transition from the pro-embryogenic mass to the embryo is a key developmental switch during Norway spruce SE that is initiated by withdrawal of plant growth regulators, leading to rapid accumulation of early somatic embryos, with a high yield of high-quality cotyledonary somatic embryos generated when ABA is added^40^. ABA regulates somatic embryo maturation in angiosperms and gymnosperms^41^. ABA is provided to the embryo by the female gametophyte in the seed. As the female gametophyte is absent *in vitro*, ABA must be provided by the medium. In most coniferous species evaluated so far, somatic embryo development must be stimulated by exogenous ABA, which concomitantly reduces cell proliferation, probably by affecting nucleotide biosynthesis. Osmotic pressure-regulating agents, such as PEG, also have a significant effect on the maturation of somatic embryos. The PEG4000 concentration (150 g/l) in the medium during Hinoki cypress SE is critical for embryo maturation^42^. Adding 3.75% PEG advances maturation by 2 weeks and changes morphometric characteristics; e.g., embryos are 30–40% longer. Medium containing 3.75% PEG is the most beneficial for embryo development of a Norway spruce genotype^43^. In our study, the highest yield of early somatic embryos (53.33 embryos/0.2 g embryogenic tissue mass) was generated from medium supplemented with 50 μmol L^−1^ ABA and 100 g L^−1^ PEG6000 during a 2-week incubation. The yield was significantly higher than the average number of early embryos calculated from the incubation results on media containing either ABA or PEG alone.

An interesting feature in this study is that Chinese fir somatic embryos can not only be produced from an embryogenic callus in maturation medium containing the appropriate concentrations of ABA and PEG600, but they also developed after continuous transfer in medium containing cytokinins as the main growth regulator. What's more, the data presented here show that induction of adventitious buds was easier than SE when immature Chinese fir embryos were cultured with cytokinins as the dominant growth regulator. Similar results have been observed in Chinese fir using explants of mature zygotic embryos^18^, and cotyledons and hypocotyls^17^; i.e., Chinese fir organogenesis occurs by means of adventitious buds. Subsequently, calluses have been produced from 7-day-old Chinese fir seedling hypocotyls cultured with cytokinins, but only a high percentage (approximate 93.73%) of adventitious buds^15^ and no somatic embryos were observed. Compared with previous studies, the obvious difference was the mode of SE. Chinese fir somatic embryos used in previous studies were all obtained by direct SE, whereas we used an indirect SE mode. Furthermore, immature dominant-stage embryos are less mature than mature embryos, cotyledons, and hypocotyls, and our using them as explants may explain why indirect SE was effective in Chinese fir cultured in cytokinins. This is also the case in *Abies nordmanniana* ^34^, as SE was easily induced from immature embryos with cytokinins as the sole plant growth regulator.

The genotype effect is crucial in plant organogenesis, particularly for SE induction^19, 44^. We found different results for Chinese fir SE based on the mother tree, suggesting a benefit of selecting the most responsive mother tree. The most responsive seed families for *Pinus radiata* SE were identified from seven genotypes of parent trees over 2 consecutive years^27^. However, several reports have pointed out that the effect of genotype or family should be interpreted carefully, as no single test includes all culture conditions, and genotypes can behave differently under different conditions^45–47^. The experimental results show that the seed families from 25 genotypes of mother trees responded significantly differently; these differences between genotypes might be reduced by changing and optimizing the culture conditions. Our data show that SE induction in Chinese fir might be under strong genetic control, and the maternal effect was an important aspect that should not be ignored. This is consistent with previous studies in *Picea glauca* ^48, 49^, *Pinus sylvestris* ^50^, and *Pinus taeda* ^51^.

In conclusion, we determined that immature cleavage polyembryony-stage embryos were at the best stage to initiate induction of embryogenic tissue via Chinese fir SE when the embryos were cultured in high concentrations of auxin/cytokinins. The reduced auxin and cytokinin concentrations during embryogenic tissue proliferation maintained the tissue in a better micro-morphological arrangement for a longer time. High quality and a massive quantity of mature somatic embryos were obtained from embryogenic tissues induced in maturation medium containing optimal concentrations of growth regulators (ABA and PEG6000). The dominant embryo developmental stage had the best embryogenic response to culture media containing cytokinins as the dominant growth regulators. Somatic embryos also developed from the embryogenic tissue obtained during the initiation process in subsequent successive transfer cultures without changing culture conditions. Finally, we identified the most responsive mother tree genotypes by demonstrating their potential to initiate embryogenesis and mature somatic embryos. Although further research is required to optimize somatic embryo proliferation and germination into plantlets, these findings could lead to tremendous labor savings and enhance maturation and conversion yield.
